# The impact of daily steps on polycystic ovary syndrome patients

**DOI:** 10.3389/fendo.2026.1744228

**Published:** 2026-04-16

**Authors:** Tianmei Wang, LiJuan Zhang, Liya Ma, Xiaodong Luo, Cong Li

**Affiliations:** 1 Department of Gynecology, The First Affiliated Hospital of Chongqing Medical University, Chongqing, China; 2 Department of Gynecology and Obstetrics, The Second Affiliated Hospital of Chongqing Medical University, Chongqing, China

**Keywords:** abdominal fat, daily steps, metabolism, PCOS, walking

## Abstract

**Objectives:**

As a reproductive-metabolic disorder, lifestyle interventions play a crucial role in managing Polycystic Ovary Syndrome (PCOS) patients. However, maintaining physical activity in PCOS patients is often challenging. Daily steps are now widely used to provide relatively simple physical activity goals for the general public. This study examines the impact of daily steps on patients with PCOS, aiming to develop simple and effective intervention strategies for them.

**Methods:**

This study included anthropometric parameters, metabolic indicators, sex hormones, and daily steps from women aged 16–40 years with PCOS (n=167) and without PCOS (n=152). Participants were divided into obese and non-obese groups based on BMI ≥ 25 kg/m² for intergroup comparisons, comprehensively evaluating the association between daily steps and various indicators.

**Results:**

Among PCOS patients, 42.9% were sedentary, while only 8.8% were active or highly active. Indicators significantly correlated with step count in the correlation analysis underwent further multivariate linear regression analysis. RCS analysis further validated the positive linear relationship between HDL and daily step count in PCOS patients (P for non-linear = 0.125). For PCOS patients without obesity, daily step count showed linear relationships with TG (P for non-linear = 0.211), HDL (P for non-linear = 0.255), and VAI (P for non-linear = 0.839). Additionally, daily steps showed linear relationships with body fat percentage (BFP, p=0.033, *R*^2^ = 0.454), android fat percentage (p=0.045, *R*^2^ = 0.413), Android/Gynoid ratio (A/G, p=0.041, *R*^2^ = 0.425), fat mass index (FMI, p=0.007, *R*^2^ = 0.671), and percent trunk fat (PTF, p=0.041, *R*^2^ = 0.427).

**Conclusion:**

Daily steps exhibits a beneficial association with lipid and body fat metabolism in PCOS patients without obesity, making it a viable indicator for quantifying physical activity that can be incorporated into PCOS patient management.

## 1 Introduction

PCOS is a heterogeneous disorder. It is characterized by hyperandrogenism, ovulatory dysfunction, poly-cystic ovarian morphology, and/or elevated Anti-Müllerian hormone (AMH) levels. This disorder affects an estimated 10–13% of women worldwide (1). In addition to reproductive symptoms, most women with PCOS exhibit metabolic disorders such as insulin resistance, hyperinsulinemia, and dyslipidemia. There is also a significantly increased risk of developing type 2 diabetes and other metabolic diseases (2). Consequently, metabolic regulation is a promising pathway for managing PCOS and reducing its burden (3).

Guidelines recommend that adults engage in at least 150–300 minutes of moderate-intensity physical activity or 75–150 minutes of vigorous-intensity physical activity per week, or more activity for greater health benefits (1, 4). However, high dropout rates have been observed in physical activity intervention studies among women with PCOS over shorter study periods, indicating significant barriers to lifestyle modification (5–7). Given the critical role of lifestyle interventions in PCOS management and the difficulty of engaging women with PCOS in lifestyle changes, there is an urgent need for a better understanding of lifestyle modification approaches for weight management in this population. Currently, daily steps, as an easily measurable and understandable metric, has been widely used to provide relatively simple physical activity goals for the public. Over the past decade, research evidence has expanded on the dose-response association between step count and health outcomes, including all-cause mortality, cardiovascular disease, and type 2 diabetes (8–10). Daily steps have consistently been associated with lower risks of major health outcomes, with risk reductions observed even at lower step levels (11). Furthermore, monitoring daily steps has become increasingly accessible with the widespread adoption of step-tracking technology in smartphones (12). While numerous studies have examined the association between daily steps and metabolic diseases across various populations, no dedicated research currently exists on the impact of daily steps specifically for PCOS patients.

Due to the widespread recognition of PCOS as a reproductive-metabolic disorder (3), understanding the impact of daily steps on PCOS patients is crucial for developing effective strategies to improve their condition. Therefore, this study innovatively investigated the correlation between daily steps and clinical measurements and biochemical indicators in PCOS patients. These findings may inform simple, easily measurable, and followable strategies to enhance the health status of PCOS patients.

## 2 Material and methods

### 2.1 Study participants

This study included 167 women with PCOS and 152 age-matched healthy control women who visited the gynecology outpatient department of the First Affiliated Hospital of Chongqing Medical University between May 2024 and March 2025 with complete data. The control group comprised women who visited for non-menstrual disorder factors such as vaginitis or underwent physical examinations. The study protocol was approved by the Institutional Review Board. All women recruited for the study were aged 16–40 years. PCOS was diagnosed according to the 2003 Rotterdam criteria, requiring at least two of the following symptoms: (1) oligomenorrhea or amenorrhea; (2) biochemical or clinical hyperandrogenism; (3) ultrasound-confirmed polycystic ovarian morphology, after other causes of these features are excluded. All control groups experienced regular menstruation each month. Participants undergoing hormone therapy, including OCP (oral contraceptive pills) or other anti-androgen medications, recent use of metabolism-altering drugs (such as glucocorticoids and metformin) within the past month, pregnancy, breastfeeding, weight changes within the past month, and cardiovascular, liver, or kidney disease, or other hormonal disorders (e.g., hyperprolactinemia, thyroid dysfunction, etc.) were excluded.

### 2.2 Measurement of clinical

All participants underwent a comprehensive clinical examination, with weight recorded to the nearest 0.1 kg and height measured with an approximation of 0.5 cm. Body mass index (BMI) was then calculated as weight divided by the square of height (kg/m²). According to the WHO-recommended BMI classification standards for Asian populations, obesity is defined as the BMI ≥ 25 kg/m² (13, 14). WC was measured in the standing position, 1 cm above the umbilicus. Hip circumference (HC) was measured at the widest point of the hip from a side view. All anthropometric measurements were performed by the same observer. Subsequently, the waist-to-hip ratio (WHR) and waist-to-height ratio (WHtR) were calculated. Then a trans-abdominal pelvic ultrasonography was done by experienced radiologists using a 3-5.5 MHz curvilinear probe in the early follicular phase of the menstrual cycle (for women with regular cycles) or a random day for those with irregular cycles.

Bioelectrical impedance analysis (BIA) offers a more affordable and practical approach to body composition analysis by leveraging differences in electrical conductivity and body composition (15). Using the InBody H20 (Seoul, Korea), it predicts body fat percentage (BFP), visceral fat level (VFL), and basal metabolic rate (BMR). Participants were instructed to stand barefoot on the device platform’s electrodes while holding the hand electrodes, following the manufacturer’s guidelines. Prior to measurement, subjects fasted, wore light clothing, and underwent assessment at room temperature.

Additionally, trained technicians used the same DXA scanner (Discovery A, Hologic, Bedford, USA) to measure body composition in 10 randomly selected PCOS patients without obesity. Measurements included BFP, lean body mass, bone mineral density (BMD), %android fat, %gynoid fat, android/gynoid ratio (A/G), visceral adipose tissue mass (VAT mass), percent trunk fat (PTF), and fat mass index (FMI = fat mass/height²).

### 2.3 Biochemical indicators

Serological testing for all participants involved drawing blood from the antecubital vein in the morning after an overnight fast of 8-12h, and on 3–5 days of the participant’s menstrual cycle (or following ultrasound confirmation of no dominant follicle in amenorrheic individuals). Blood samples were used to determine participants’ total testosterone (TT), luteinizing hormone (LH), follicle-stimulating hormone (FSH), fasting plasma glucose (FPG), fasting insulin (FINS), vitamin D (VD), and lipid levels, including total cholesterol (TC), triglycerides (TG), high-density lipoprotein (HDL), and low-density lipoprotein (LDL). Based on these data, we calculated the homeostasis model assessment-estimated insulin resistance (HOMA-IR) using the formula: FPG× FINS/22.5 (16). The formula for calculating the visceral adiposity index (VAI) is (17)

(1) $$\text{VAI}=(\frac{\text{WC}}{36.58+(1.89\times \text{BMI})})\times (\frac{\text{TG}}{0.81})\times (\frac{1.52}{\text{HDL}})$$

Using (WC-58)*TG to calculate the lipid accumulation product (LAP)(18).

### 2.4 Assessment of daily steps

WeChat is one of the most widely used social media platforms, boasting approximately 1.1 billion active users in China. It can automatically record step count data via sensors built into mobile phones (19). We tracked the daily step counts of participants using WeChat on their phones for the 7 days preceding their clinic visit and calculated the average. Participants carried smartphones for ≥ 10 hours daily, which is the standard practice for measuring daily walking Tudor-Locke (20). Based on Tudor-Locke et al.’s classification of physical activity by daily step count: (i) *<*5000 steps/day is classified as “sedentary lifestyle index”; (ii) 5000–7499 steps/day be considered “low active”; (iii) 7500–9999 steps/day were classified as “somewhat active”; (iv) ≥ 10,000 steps/day indicates “active.” Individuals walking >12,500 steps daily may be categorized as “highly active” (21).

### 2.5 Statistical analysis

All statistical analyses were performed using SPSS Statistics software (version 26.0) and R program (version 4.5.1). A two-sided p-value <0.05 was considered statistically significant. Measurements following a normal distribution are described as $\bar{x}\;$ ± s; independent t-tests are used, with Welch’s t-test employed when variances are unequal. Those not following a normal distribution are described as median (min, max) and analyzed using the Mann-Whitney U test. Additionally, bivariate Spearman correlation analysis was employed to evaluate correlations between continuous variables. Multivariate linear regression was applied to assess the independent association between daily step count and these clinical indicators, controlling for age and BMI as potential confounders. Restricted cubic splines (RCS) using the “rms” package were utilized to examine nonlinear associations between daily steps and clinical indicators. To provide sufficient flexibility to capture potential nonlinear trends while avoiding overfitting, the knots were located at the 5th, 35th, 65th, and 95th percentiles of the daily steps distribution.

## 3 Results

### 3.1 Characteristics of the study sample

**Figure 1:**
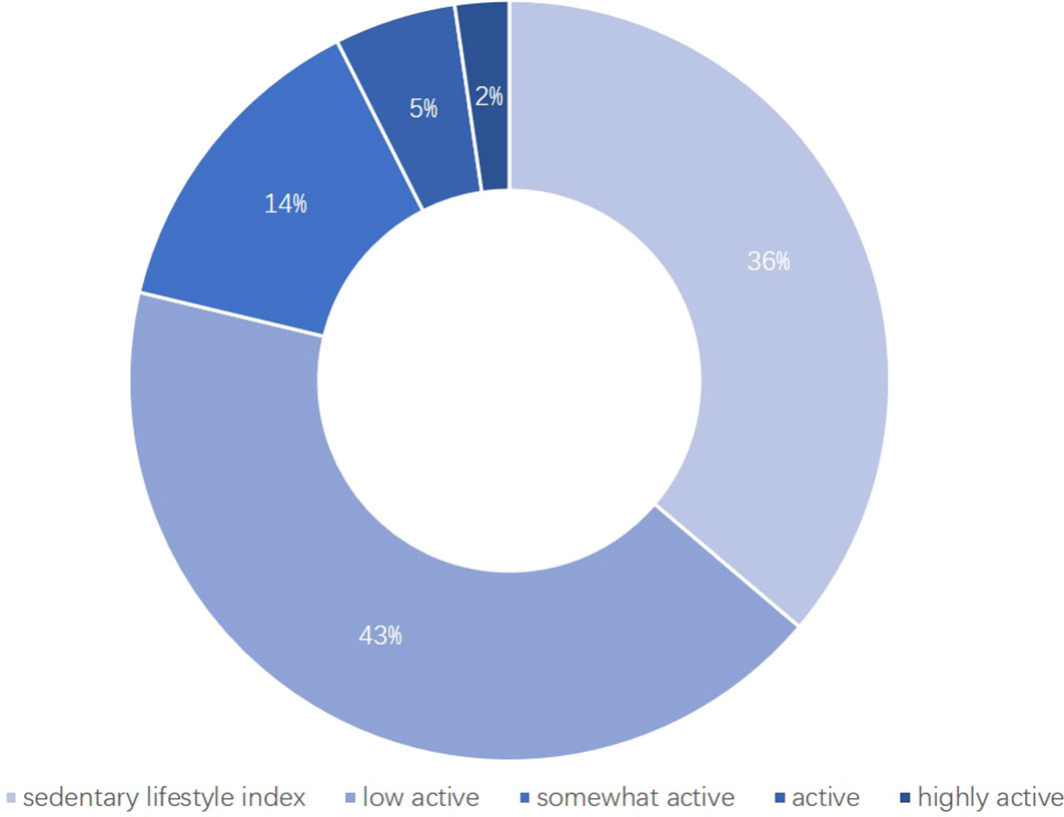
Physical activity classification for PCOS patients.

The baseline characteristics of study participants are shown in **Supplementary Table 1**. This study included 167 PCOS patients and 152 control participants. The obesity rate among PCOS patients (52/167, 31.14%) was significantly higher than that in the control group (23/152, 15.13%). Compared to controls, PCOS patients without obesity exhibited higher levels of TC, LDL, FPG, HOMA-IR, VAI, BFP, and BMR, along with lower HDL levels. These differences were not evident in patients with obesity. **Figure 1** shows the percentage distribution of PCOS participants categorized by physical activity levels based on daily step counts. 42.9% of patients were classified as sedentary, 32.0% as low active, 16.3% as somewhat active, 6.1% as active, and only 2.7% as highly active.

### 3.2 Correlation between daily steps and clinical measurements or biochemical parameters

**Table 1** presents the bivariate associations between daily steps and various clinical measurements and biochemical indicators in PCOS patients. For all PCOS study participants, daily steps was positively correlated with TG (r = -0.201, p = 0.045) and VAI (r = -0.245, p = 0.015), and negatively correlated with HDL (r = 0.290, p = 0.003). Among PCOS patients without obesity, daily steps correlated positively with WC (r = -0.231, p = 0.018), TG (r = -0.318, p = 0.008), FINS (r = -0.275, p = 0.022), HOMA-IR (r = -0.248, p = 0.042), VAI (r = -0.383, p = 0.001), BFP (r = -0.246, p = 0.012), and VFL (r = -0.226, p=0.022), and positively correlated with HDL (r=0.379, p=0.001). Daily steps in PCOS patients with obesity showed no statistically significant correlation with any of these indicators.

**Table 1:** Correlation between daily steps and composition parameters and biochemical parameters in PCOS patients.

Variable	PCOS		Non-obese PCOS		Obese PCOS	
	r	p	r	p	r	p
BMI (kg/m²)	0.036	0.661	-0.126	0.197	-0.057	0.723
WC (cm)	-0.028	0.733	$-0.231^{*}$	0.018	-0.028	0.861
HC (cm)	-0.041	0.624	-0.172	0.080	-0.295	0.061
WHR	0.029	0.726	-0.130	0.185	0.097	0.545
WHtR	0.010	0.909	-0.190	0.053	0.115	0.474
TC (mmol/L)	0.008	0.935	-0.109	0.375	0.251	0.165
TG (mmol/L)	$-0.201^{*}$	0.045	$-0.318^{**}$	0.008	-0.048	0.796
LDL (mmol/L)	-0.052	0.609	-0.174	0.157	0.190	0.297
HDL (mmol/L)	$0.290^{**}$	0.003	$0.379^{**}$	0.001	0.158	0.389
FPG (mmol/L)	-0.062	0.532	-0.115	0.339	0.057	0.749
FINS (uIU/ml)	-0.164	0.102	$-0.275^{*}$	0.022	-0.087	0.638
HOMA-IR	-0.140	0.165	$-0.248^{*}$	0.042	-0.040	0.828
VD (ng/ml)	0.004	0.973	0.102	0.435	-0.234	0.240
LH/FSH	-0.047	0.585	0.003	0.979	-0.117	0.486
TT (nmol/L)	-0.020	0.813	0.022	0.831	-0.106	0.530
VAI	$-0.245^{*}$	0.015	$-0.383^{**}$	0.001	-0.038	0.837
LAP	-0.098	0.332	-0.211	0.086	-0.049	0.792
BFP(%)	-0.096	0.253	$-0.246^{*}$	0.012	-0.212	0.184
VFL	-0.109	0.195	$-0.226^{*}$	0.022	-0.262	0.098
BMR (kcal)	0.077	0.358	-0.006	0.951	-0.110	0.493

^∗^*p <* 0.05, ^∗∗^*p <* 0.01, BMI, Body Mass Index; WC, Waist Circumference; HC, Waist Circumference; WHR, Waist-to-Hip Ratio; WHtR, Waist-to-Height Ratio; TC, Total Cholesterol; TG, Triglycerides; LDL, Low-Density Lipoprotein; HDL, High-Density Lipoprotein; FPG, Fasting Plasma Glucose; FINS, Fasting Insulin; HOMA-IR, Homeostatic Model Assessment of Insulin Resistance; VD, Vitamin D; LH/FSH, Luteinizing Hormone/Follicle-Stimulating Hormone; TT, Total Testosterone; VAI, Visceral Adiposity Index; LAP, Lipid Accumulation Product; BFP, Body Fat Percentage; VFL, Visceral Fat Level; BMR, Basal Metabolic Rate.

### 3.3 Regression analysis of daily steps and related parameters in PCOS patients

**Table 2** presents the results of multivariate linear regression analysis examining daily steps and the aforementioned indicators among PCOS patients across groups. After adjusting for BMI and age variables, overall daily step count in PCOS patients remained significantly correlated with HDL (R²=0.452, B = 0.00006, p*<*0.001) and VAI (R²=0.355, B=-0.00008, p=0.042). Among PCOS patients without obesity, daily step count was associated with waist circumference (R²=0.508, B=-0.0003, p=0.044), TG (R²=0.133, B=-0.00004, p=0.044), HDL (R²=0.378, B = 0.00006, p*<*0.001), VAI (R²=0.282, B=-0.00009, p=0.005), and BFP (R²=0.423, B = 0.0004, p=0.042).

**Table 2:** Multivariate linear regression analysis of daily steps and related parameters in PCOS patients.

Variable	B (95% CI)	p	R²
PCOS			
TG (mmol/L)			
Age	0.035 (0.005, 0.065)	0.024	0.202
BMI	0.066 (0.034, 0.097)	*<*0.001	
Daily steps	-0.00003 (*>*-0.001, *<*0.001)	0.223	
HDL (mmol/L)			
Age	-0.006 (-0.019, 0.007)	0.340	0.452
BMI	-0.048 (-0.062, -0.034)	*<*0.001	
Daily steps	0.00006 (*<*0.001, *<*0.001)	¡0.001	
VAI			
Age	0.083 (0.033, 0.134)	0.001	0.355
BMI	0.162 (0.108, 0.216)	*<*0.001	
Daily steps	-0.00008 (*>*-0.001, *>*-0.001)	0.042	
Non-obese PCOS			
WC (cm)			
Age	0.181 (-0.037, 0.399)	0.102	0.508
BMI	2.068 (1.619, 2.517)	*<*0.001	
Daily steps	-0.0003 (-0.001, *<*0.001)	0.044	
TG (mmol/L)			
Age	0.016 (-0.012, 0.043)	0.271	0.133
BMI	0.048 (-0.010, 0.106)	0.101	
Daily steps	-0.00004 (*>*-0.001, *>*-0.001)	0.044	
HDL (mmol/L)			
Age	0.003 (-0.015, 0.022)	0.741	0.378
BMI	-0.076 (-0.114, -0.038)	*<*0.001	
Daily steps	0.00006 (*<*0.001, *<*0.001)	*<*0.001	
FINS (uIU/ml)			
Age	-0.139 (-0.333, 0.055)	0.157	0.223
BMI	0.650 (0.251, 1.049)	0.002	
Daily steps	-0.0003 (-0.001, *<*0.001)	0.058	
HOMA-IR			
Age	-0.036 (-0.086, 0.015)	0.162	0.252
BMI	0.206 (0.101, 0.310)	*<*0.001	
Daily steps	-0.00005 (*>*-0.001, *<*0.001)	0.149	
VAI			
Age	0.036 (-0.003, 0.076)	0.070	0.282
BMI	0.119 (0.038, 0.201)	0.005	
Daily steps	-0.00009 (*>*-0.001, *>*-0.001)	0.005	
BFP (%)			
Age	-0.088 (0.485, -0.337)	0.485	0.423
BMI	2.007 (1.494, 2.520)	*<*0.001	
Daily steps	-0.0004 (-0.001, *>*-0.001)	0.042	
VFL			
Age	-0.003 (-0.112, 0.106)	0.961	0.262
BMI	0.625 (0.400, 0.849)	*<*0.001	
Daily steps	-0.00009 (*>*-0.001, *<*0.001)	0.282	

B, Unstandardized Coefficient B; CI, Confidence Interval; BMI, Body Mass Index; WC, Waist Circumference; TG, Triglycerides; HDL, High-Density Lipoprotein; FINS, Fasting Insulin; HOMA-IR, Homeostatic Model Assessment of Insulin Resistance; VAI, Visceral Adiposity Index; BFP, Body Fat Percentage; VFL, Visceral Fat Level.

**Figure 2:**
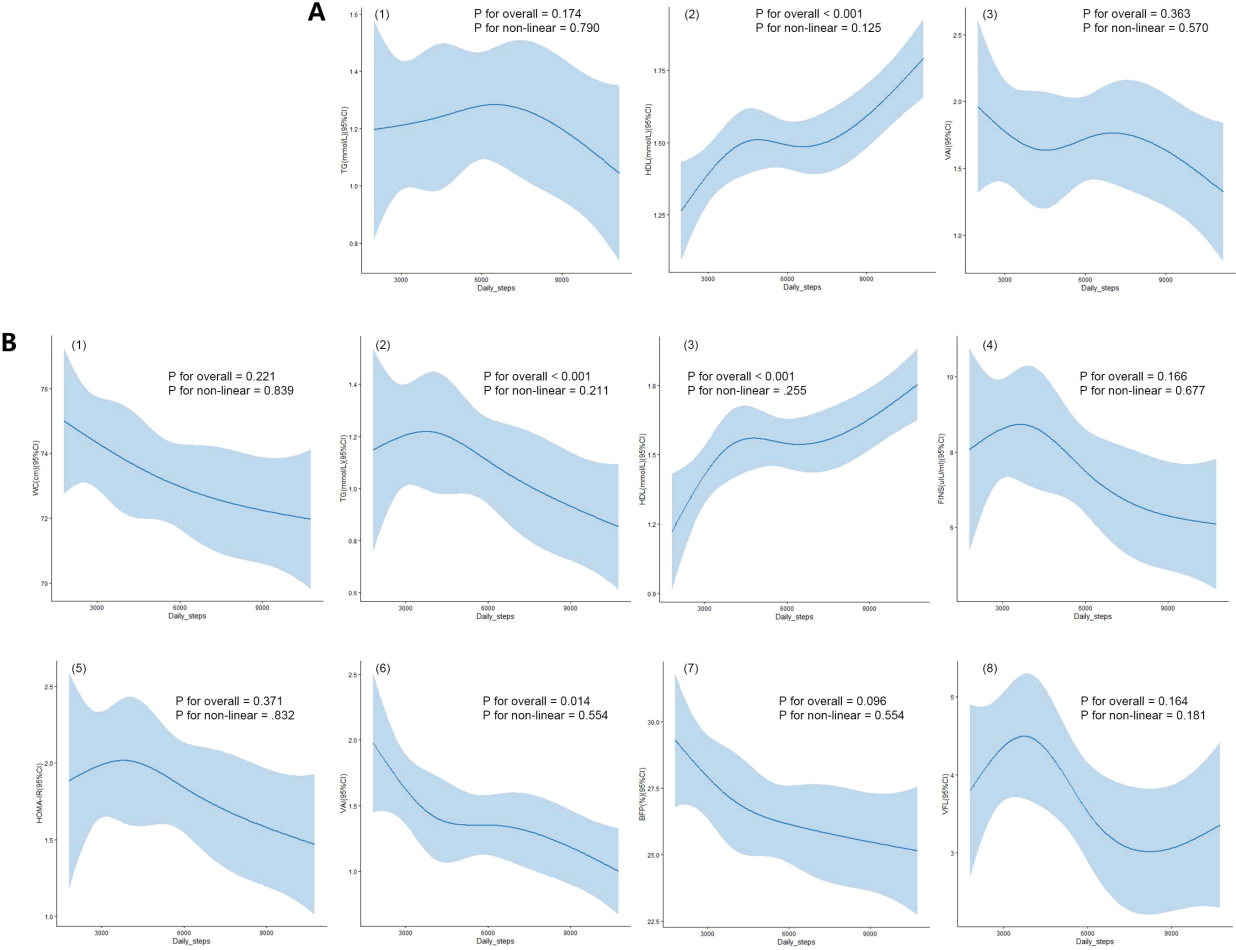
Using RCS curve analysis to examine the nonlinear relationship between daily step count and related parameters. **(A)** (1-3) Associations between TG, HDL, and VAI with daily steps in PCOS patients. **(B)** (1-8) Associations between WC, TG, HDL, FINS, HOMA-IR, VAI, BFP, and VFL with daily steps in PCOS patients without obesity. Blue shaded areas represent the 95% confidence intervals. TG, Triglycerides; HDL, High-Density Lipoprotein; VAI, Visceral Adiposity Index; WC, Waist Circumference; FINS, Fasting Insulin; HOMA-IR, Homeostatic Model Assessment of Insulin Resistance; BFP, Body Fat Percentage; VFL, Visceral Fat Level.

To investigate potential nonlinear relationships, we employed RCS fitting while controlling for age and BMI as covariates. Restricted cubic spline curves revealed a positive linear correlation between HDL and daily steps for the overall PCOS cohort (**Figure 2A**(2), p for overall <0.001, p for non-linear = 0.125). For PCOS patients without obesity, daily step count correlated with TG (**Figure 2B**(2), p for overall <0.001, p for non-linear = 0.211), HDL (**Figure 2B**(3), p for overall <0.001, p for non-linear = 0.255), and VAI (**Figure 2B**(6), p for overall = 0.035, p for non-linear = 0.839).

### 3.4 Regression analysis of daily steps and body composition in PCOS patients without obesity

**Figure 3:**
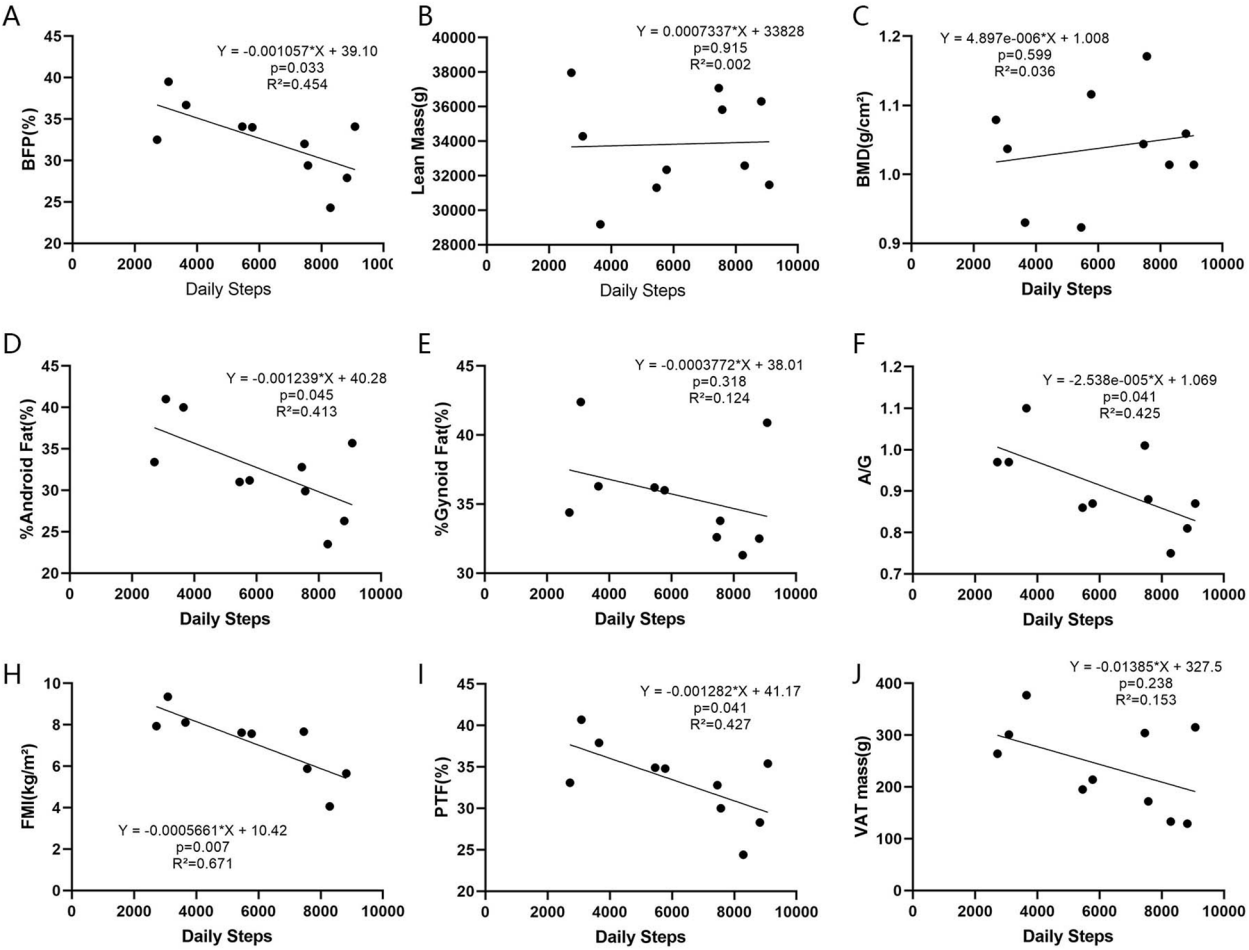
Linear regression analysis between daily steps and body composition. **(A-J)** Associations between BFP, lean mass, BMD, %Android fat, %Gynoid fat, A/G, FMI, PTF, VAT mass, and daily steps in PCOS patients without obesity. BFP, Body Fat Percentage; BMD, Bone Mineral Density; A/G, Android fat/Gynoid fat; FMI, Fat Mass Index; PTF, Percent Trunk Fat; VAT, Visceral Adipose Tissue.

**Figure 3** shows that daily steps in patients without obesity was significantly negatively correlated with BFP (p=0.033, R²=0.454, **Figure 3A**), %Android fat (p=0.045, R²=0.413, **Figure 3D**), A/G (p=0.041, R²=0.425, **Figure 3F**), FMI (p=0.007, R²=0.671, **Figure 3H**), and PTF (p=0.041, R²=0.427, **Figure 3I**). For every 1000-step increase, BFP decreased by 1.06%, %android fat decreased by 1.24%, A/G decreased by 0.03, FMI decreased by 0.57kg/m², and PTF decreased by 1.28%.

## 4 Discussion

This study is the first to investigate the association between daily steps and clinical/biochemical indicators in PCOS patients. We found that for PCOS patients, particularly non-obese individuals, increased daily step count correlates with elevated HDL levels, with each 1000-step increase corresponding to a 0.06 mmol/L rise in HDL. Additionally, for PCOS patients without obesity, increased daily step count linearly correlates with reduced TG and VAI levels. For every 1,000-step increase in daily step count, TG decreased by 0.04 mmol/L, VAI decreased by 0.09, BFP decreased by 1.06%, android fat percentage decreased by 1.24%, A/G ratio decreased by 0.03, FMI decreased by 0.57 kg/m*R*^2^, and PTF decreased by 1.28%. By establishing specific dose-dependent associations, higher daily step counts were found to be beneficial for PCOS patients, particularly non-obese individuals.

Compared to women without PCOS, those with PCOS are more sedentary and less active (22). Among participants in our study, PCOS patients—regardless of obesity status—had a median daily step count below 6,000 steps. According to Tudor-Locke et al.’s physical activity classification, nearly half (42.9%) of PCOS patients were sedentary, while only 8.8% were active or highly active. Previous studies have consistently shown that increased daily steps can reduce BMI, WC, and BFP (23, 24). Similar findings were observed in PCOS patients without obesity in this study. Linear regression analysis revealed a significant negative correlation between daily steps and WC (B = -0.145) and BFP (B = -0.160) in PCOS patients without obesity. Although RCS analysis failed to detect a significant relationship, a clear negative trend was still discernible, potentially due to insufficient sample size resulting in overly broad confidence intervals. Concurrently, results for patients with obesity were inconclusive. This may stem partly from the smaller sample size and partly from the fact that guidelines recommend adults engage in at least 250 minutes of moderate-intensity activity or 150 minutes of vigorous-intensity activity per week, or an equivalent combination of both, to moderately reduce body weight and prevent weight regain (1). Based on existing literature, clinically significant weight loss is unlikely to be achieved unless the total volume of aerobic exercise training is exceptionally high Swift et al. (25). Although research has found that various types of exercise can improve insulin sensitivity, intensity and duration are crucial Houmard et al. (26). Thus, walking activity may yield limited effects for patients with obesity, who likely require more intense exercise.

Additionally, our research found that increased daily step count impacts metabolism in PCOS patients. In a study by Sisson involving 1,446 adults, those with higher daily step counts tended to have lower prevalence of metabolic syndrome, higher HDL levels, and lower TG levels. For every additional 1,000 steps per day, the prevalence of metabolic syndrome decreased by 10% (27). In our study, for PCOS patients overall, each 1,000-step increase in daily step count was associated with a 0.06 mmol/L increase in HDL. For patients without obesity, each 1,000-step increase was linked to a 0.04 mmol/L decrease in TG. These results were further validated in the RCS analysis. Additionally, while FINS and HOMA-IR correlated with daily steps in the correlation analysis, subsequent multivariate regression indicated BMI as the significant predictor, independent of step count. This suggests that BMI may act as a mediating factor in the relationship between daily step count and insulin resistance. However, no significant correlation was found between daily step count and BMI in the correlation analysis, larger sample sizes may be required in future studies to detect potential small effects.

In our study, while VFL was only associated with BMI, the results for VAI were simultaneously influenced by daily steps. For every 1,000-step increase in daily activity, VAI decreased by 0.09. Introduced in 2010, VAI has been demonstrated to effectively assess visceral fat distribution and dysfunction in adults. It is considered a useful alternative to visceral CT scans for identifying individuals at higher risk of metabolic disorders associated with visceral obesity, including insulin resistance, dyslipidemia, and cardiovascular risk factors (17). Studies have found VAI and WC to be strong predictors of PCOS (AUC, 98% CI [1.00–1.00]) (28). Furthermore, PCOS patients with the oligomenorrhea phenotype exhibit higher VAI scores than controls (29). Small-sample studies have also confirmed significant correlations between VAI and metabolic syndrome markers in PCOS women without obesity, suggesting its potential as a screening tool for cardiometabolic risk (30, 31). However, other research found no significant alterations in VAI among PCOS patients without obesity (32). Additionally, DXA body composition results in PCOS patients without obesity with small sample sizes showed a significant negative linear relationship with daily steps, including BFP, %android fat, A/G, FMI, and PTF. Therefore, increasing daily steps may potentially improve the central obesity trend in patients without obesity.

DXA is now widely used in body composition research across various diseases Shepherd et al. (33), but its application in the clinical diagnosis and management of PCOS remains limited. Consequently, the sample size obtained in this retrospective study was constrained, serving only as a preliminary exploration. In this study, DXA body composition results from a small sample of PCOS patients without obesity showed a significant negative linear relationship with daily step count, including BFP, android fat percentage, A/G ratio, FMI, and PTF—metrics reflecting overall and central fat distribution. Current research on exercise effects in patients without obesity is limited. One study involving 30 PCOS patients without obesity suggested that short-term structured exercise significantly reduced waist and hip circumferences Turan et al. (34). Thus, our findings provide evidence that increased physical activity may potentially improve the trend toward central obesity in patients without obesity.

This study innovatively incorporates daily step count into PCOS management. Compared to existing exercise regimens, increasing daily steps is easier to measure and more sustainable. However, previous assessments of physical activity and sedentary behavior have largely relied on questionnaires completed by participants through recall, resulting in limited objectivity Tudor-Locke et al. (35). Passive data collection via the WeChat platform enables convenient and rapid long-term monitoring while minimizing participant burden, and yields relatively objective results. Previous research has validated the accuracy of daily step counts collected through WeChat (12, 19). We required participants to carry their smartphones for at least 10 hours per day, thereby minimizing data errors caused by inconsistent phone use and ensuring data quality. Second, prior research indicates that three days of pedometer data can provide an effective estimate of habitual activity (36). Collecting 7 days of 45 data, encompassing both weekdays and weekends, provides a representative snapshot of participants’ physical activity patterns.

However, this study has several limitations. First, current research is constrained by its cross-sectional nature, making it impossible to determine the temporal sequence and directionality of this association or to establish causality. The logical sequence remains to be further validated. And sample size limitations may exist, particularly regarding the smaller number of participants with obesity. Second, smartphone-based data may underestimate step counts, particularly during indoor activities without phone access. Nevertheless, a prior study reported that Chinese citizens wear smartphones for an average of over 13 hours daily, suggesting most walking activities should be captured (37). Third, despite adjusting analyses for key confounders, residual confounding cannot be entirely ruled out.

## 5 Conclusion

Our findings indicate that daily steps exhibits a linear, beneficial association with lipid profiles and body fat parameters in PCOS patients, particularly individuals without obesity. This includes TG, HDL, VAI, BFP, %android fat, A/G, FMI, and PTF. These results suggest that daily steps can be incorporated into the management of PCOS patients as a metric for quantifying physical activity. 

## Data Availability

The raw data supporting the conclusions of this article will be made available by the authors, without undue reservation.

## References

[1] Teede HJ Tay CT Laven JJE Dokras A Moran LJ Piltonen TT . Recommendations from the 2023 international evidence-based guideline for the assessment and management of polycystic ovary syndrome. Eur J Endocrinol. (2023) 189:G43–64. doi: 10.1093/ejendo/lvad096. PMID: 37580861

[2] Glueck CJ Goldenberg N . Characteristics of obesity in polycystic ovary syndrome: Etiology, treatment, and genetics. Metab Clin Exp. (2019) 92:108–20. doi: 10.1016/j.metabol.2018.11.002. PMID: 30445140

[3] Zhang Y Chen Z-J Zhao H . Polycystic ovary syndrome: A metabolic disorder with therapeutic opportunities. Cell Metab. (2025) 37:1932–49. doi: 10.1016/j.cmet.2025.08.002. PMID: 40912248

[4] WHO . WHO Guidelines on Physical Activity and Sedentary Behaviour. Geneva, Switzerland: World Health Organization (2020).

[5] Harrison CL Lombard CB Moran LJ Teede HJ . Exercise therapy in polycystic ovary syndrome: A systematic review. Hum Reprod Update. (2011) 17:171–83. doi: 10.1093/humupd/dmq045. PMID: 20833639

[6] Hutchison SK Stepto NK Harrison CL Moran LJ Strauss BJ Teede HJ . Effects of exercise on insulin resistance and body composition in overweight and obese women with and without polycystic ovary syndrome. J Clin Endocrinol Metab. (2011) 96:E48–56. doi: 10.1210/jc.2010-0828. PMID: 20926534

[7] Thomson RL Buckley JD Lim SS Noakes M Clifton PM Norman RJ . Lifestyle management improves quality of life and depression in overweight and obese women with polycystic ovary syndrome. Fertil. Steril. (2010) 94:1812–6. doi: 10.1016/j.fertnstert.2009.11.001. PMID: 20004371

[8] Del Pozo Cruz B Gallardo-Gomez D Del Pozo-Cruz J Ding D . How many steps a day to reduce the risk of all-cause mortality? A dose-response meta-analysis. J Internal Med. (2022) 291:519–21. doi: 10.1111/joim.13413. PMID: 34808011

[9] Sheng M Yang J Bao M Chen T Cai R Zhang N . The relationships between step count and all-cause mortality and cardiovascular events: A dose-response meta-analysis. J Sport. Health Sci. (2021) 10:620–8. doi: 10.1016/j.jshs.2021.09.004. PMID: 34547483 PMC8724621

[10] Garduno AC LaCroix AZ LaMonte MJ Dunstan DW Evenson KR Wang G . Associations of daily steps and step intensity with incident diabetes in a prospective cohort study of older women: The OPACH study. Diabetes Care. (2022) 45:339–47. doi: 10.2337/dc21-1202. PMID: 35050362 PMC8914434

[11] Ding D Nguyen B Nau T Luo M Del Pozo Cruz B Dempsey PC . Daily steps and health outcomes in adults: A systematic review and dose-response meta-analysis. Lancet Public Health. (2025) 10:e668–81. doi: 10.1016/S2468-2667(25)00164-1. PMID: 40713949

[12] Zhang W Mu Q Chen P He Y Zhou Y Fang J . Associations of daily step counts with depressive symptoms during pregnancy: Ruian birth cohort study. BMC Public Health. (2025) 25:2849. doi: 10.1186/s12889-025-24181-2. PMID: 40830780 PMC12366126

[13] WHO . Obesity and overweight. In: World Health Organization Fact Sheet. Geneva: World Health Organization. (2025). Available online at: https://www.who.int/news-room/fact-sheets/detail/obesity-and-overweight (Accessed April 1, 2026).

[14] Rotterdam ESHRE/ASRM-Sponsored PCOS consensus workshop group . Revised 2003 consensus on diagnostic criteria and long-term health risks related to polycystic ovary syndrome (PCOS). Hum Reprod. (2004) 19:41–7. doi: 10.1093/humrep/deh098. PMID: 14688154

[15] Ferguson CE Lambell KJ . Clinimetrics: Bioelectrical impedance analysis in clinical practice. J Physiother. (2022) 68:280. doi: 10.1016/j.jphys.2022.05.007. PMID: 35715376

[16] Matthews DR Hosker JP Rudenski AS Naylor BA Treacher DF Turner RC . Homeostasis model assessment: Insulin resistance and beta-cell function from fasting plasma glucose and insulin concentrations in man. Diabetologia. (1985) 28:412–9. doi: 10.1007/BF00280883. PMID: 3899825

[17] Amato MC Giordano C Galia M Criscimanna A Vitabile S Midiri M . Visceral adiposity index: A reliable indicator of visceral fat function associated with cardiometabolic risk. Diabetes Care. (2010) 33:920–2. doi: 10.2337/dc09-1825. PMID: 20067971 PMC2845052

[18] Kahn HS . The “lipid accumulation product” performs better than the body mass index for recognizing cardiovascular risk: A population-based comparison. BMC Cardiovasc Disord. (2005) 5:26. doi: 10.1186/1471-2261-5-26, PMID: 16150143 PMC1236917

[19] Wang Y Zhang Y Bennell K White DK Wei J Wu Z . Physical distancing measures and walking activity in middle-aged and older residents in Changsha, China, during the COVID-19 epidemic period: Longitudinal observational study. J Med Internet Res. (2020) 22:e21632. doi: 10.2196/21632. PMID: 33027035 PMC7592463

[20] Tudor-Locke C . A catalog of rules, variables, and definitions applied to accelerometer data in the National Health and Nutrition Examination Survey 2003–2006. Prevent. Chronic. Dis. (2012) 9:4. doi: 10.5888/pcd9.110332. PMID: 22698174 PMC3457743

[21] Tudor-Locke C Bassett DR . How many steps/day are enough? Preliminary pedometer indices for public health. Sport. Med. (2004) 34:1–8. doi: 10.2165/00007256-200434010-00001. PMID: 14715035

[22] Moran LJ Ranasinha S Zoungas S McNaughton SA Brown WJ Teede HJ . The contribution of diet, physical activity and sedentary behaviour to body mass index in women with and without polycystic ovary syndrome. Hum Reprod. (2013) 28:2276–83. doi: 10.1093/humrep/det256. PMID: 23771201

[23] Cook I Alberts M Lambert EV . Relationship between adiposity and pedometer-assessed ambulatory activity in adult, rural African women. Int J Obes. (2008) 32:1327–30. doi: 10.1038/ijo.2008.26. PMID: 18332881

[24] Dwyer T Hosmer D Hosmer T Venn AJ Blizzard CL Granger RH . The inverse relationship between number of steps per day and obesity in a population-based sample: The AusDiab study. Int J Obes. (2007) 31:797–804. doi: 10.1038/sj.ijo.0803472. PMID: 17047641

[25] Swift DL Johannsen NM Lavie CJ Earnest CP Church TS . The role of exercise and physical activity in weight loss and maintenance. Prog Cardiovasc Dis. (2014) 56:441–7. doi: 10.1016/j.pcad.2013.09.012. PMID: 24438736 PMC3925973

[26] Houmard JA Tanner CJ Slentz CA Duscha BD McCartney JS Kraus WE . Effect of the volume and intensity of exercise training on insulin sensitivity. J Appl Physiol. (2004) 96:101–6. doi: 10.1152/japplphysiol.00707.2003. PMID: 12972442

[27] Sisson SB Camhi SM Church TS Tudor-Locke C Johnson WD Katzmarzyk PT . Accelerometer-determined steps/day and metabolic syndrome. Am J Prev Med. (2010) 38:575–82. doi: 10.1016/j.amepre.2010.02.015. PMID: 20494233

[28] Çakır Biçer N Ermiş AA Baş D . The role of different methods in defining cardiometabolic risk and metabolic syndrome in women with polycystic ovary syndrome. Life. (2023) 13:1959. doi: 10.3390/life13101959. PMID: 37895341 PMC10608420

[29] Amato MC Verghi M Galluzzo A Giordano C . The oligomenorrhoic phenotypes of polycystic ovary syndrome are characterized by a high visceral adiposity index: A likely condition of cardiometabolic risk. Hum Reprod. (2011) 26:1486–94. doi: 10.1093/humrep/der088. PMID: 21447694

[30] Bir A Ghosh A Chowdhury S . Visceral adiposity index and lipid accumulation product index: The promising role in assessing cardiometabolic risk in non-obese patients of PCOS. J Educ Health Promot. (2023) 12:148. doi: 10.4103/jehp.jehp_1605_22, PMID: 37404927 PMC10317285

[31] De Medeiros SF De Medeiros MAS Barbosa BB Yamamoto MMW . The role of visceral adiposity index as predictor of metabolic syndrome in obese and nonobese women with polycystic ovary syndrome. Metab Syndr. Relate. Disord. (2021) 19:18–25. doi: 10.1089/met.2020.0045. PMID: 32845813

[32] Durmus U Duran C Ecirli S . Visceral adiposity index levels in overweight and/or obese, and non-obese patients with polycystic ovary syndrome and its relationship with metabolic and inflammatory parameters. J Endocrinol Invest. (2017) 40:487–97. doi: 10.1007/s40618-016-0582-x. PMID: 27838846

[33] Shepherd JA Ng BK Sommer MJ Heymsfield SB . Body composition by DXA. Bone. (2017) 104:101–5. doi: 10.1016/j.bone.2017.06.010. PMID: 28625918 PMC5659281

[34] Turan V Mutlu EK Solmaz U Ekin A Tosun O Tosun G . Benefits of short-term structured exercise in non-overweight women with polycystic ovary syndrome: A prospective randomized controlled study. J Phys Ther Sci. (2015) 27:2293–7. doi: 10.1589/jpts.27.2293. PMID: 26311969 PMC4540866

[35] Tudor-Locke C Craig CL Brown WJ Clemes SA De Cocker K Giles-Corti B . How many steps/day are enough? For adults. Int J Behav Nutr Phys Act. (2011) 8:79. doi: 10.1186/1479-5868-8-79. PMID: 21798015 PMC3197470

[36] Newton RL Han H Johnson WD Hickson DA Church TS Taylor HA . Steps/day and metabolic syndrome in African American adults: The Jackson Heart Study. Prev Med. (2013) 57:855–9. doi: 10.1016/j.ypmed.2013.09.018. PMID: 24096141 PMC4001862

[37] Althoff T Sosič R Hicks JL King AC Delp SL Leskovec J . Large-scale physical activity data reveal worldwide activity inequality. Nature. (2017) 547:336–9. doi: 10.1038/nature23018. PMID: 28693034 PMC5774986

