# Supplementary material for: The impact of daily steps on polycystic ovary syndrome patients
Source: Front Endocrinol (Lausanne). 2026 Apr 16;17:1744228. doi: 10.3389/fendo.2026.1744228 (PMC13128351; doi:10.3389/fendo.2026.1744228)
Supplement: Supplementary file 1 [file Table1.docx]

Table S1. Basic characteristics of study participants

|  |  |  |  | Non-obese |  |  | obese |  |  |
| --- | --- | --- | --- | --- | --- | --- | --- | --- | --- |
|  | PCOS（n=167） | Control（n=152） | p | PCOS (n=115) | Control (n=129) | p | PCOS (n=52) | Control (n=23) | p |
| Age(years) | 24.42±4.51 | 25.16±3.77 | 0.445* | 24.13±4.27 | 25.17±3.77 | 0.193 | 25.06±4.98 | 25.12±3.90 | 0.703 |
| BMI(kg/m²) | 23.60±4.22 | 21.27±3.91 | ＜0.001 | 21.23±2.08 | 19.91±2.36 | ＜0.001* | 28.75±2.91 | 28.34±2.46 | 0.746 |
| WC(cm) | 79.26±11.07 | 75.20±10.60 | ＜0.001* | 73.53±6.59 | 72.04±6.54 | 0.081 | 91.72±8.20 | 92.18±9.64 | 0.832 |
| HC(cm) | 93.47±8.05 | 90.80±7.86 | 0.008 | 89.56±5.34 | 88.36±5.59 | 0.125 | 101.96±6.19 | 103.41±5.47 | 0.392 |
| WHR | 0.85±0.07 | 0.83±0.06 | 0.017 | 0.82±0.05 | 0.81±0.05 | 0.318 | 0.90±0.06 | 0.89±0.08 | 0.660 |
| WHtR | 0.50±0.07 | 0.47±0.06 | ＜0.001* | 0.46±0.04 | 0.45±0.05 | 0.124 | 0.57±0.05 | 0.57±0.05 | 0.692 |
| TC(mmol/L) | 4.70±0.86 | 4.34±0.77 | 0.004 | 4.71±0.79 | 4.26±0.69 | 0.001 | 4.68±0.99 | 4.78±1.08 | 0.775 |
| TG(mmol/L)# | 0.98（0.34,4.76） | 0.87（0.42,3.13） | 0.011 | 1.03（0.34，3.19） | 0.90（0.42，2.79） | 0.061 | 1.34（0.57,4.76） | 1.53（0.59,3.13） | 0.598 |
| LDL(mmol/L) | 2.90±0.74 | 2.58±0.70 | 0.003 | 2.89±0.61 | 2.48±0.58 | ＜0.001 | 2.94±0.93 | 3.16±1.01 | 0.496 |
| HDL(mmol/L) | 1.48±0.39 | 1.81±0.38 | ＜0.001 | 1.59±0.40 | 1.88±0.33 | ＜0.001 | 1.28±0.27 | 1.21±0.24 | 0.348 |
| FPG(mmol/L) | 5.23±0.42 | 5.01±0.44 | 0.014 | 5.17±0.36 | 4.99±0.47 | 0.047 | 5.33±0.51 | 5.11±0.30 | 0.294 |
| FINS (uIU/ml)# | 8.90（1.60,65.12） | 5.25（1.77,14.80） | 0.009 | 7.57（1.60，16.23） | 5.39（1.77，14.51） | 0.058 | 13.84（3.90,65.12） | 12.74（8.81,14.80） | 0.587 |
| HOMA-IR# | 2.11（0.33,15.14） | 1.34（0.32,5.13） | 0.008 | 1.80（0.33，4.74） | 1.28（0.32，3.55） | 0.039 | 4.38（0.87,15.14） | 3.31（2.04,5.13） | 0.967 |
| VD(ng/ml) | 15.80±5.96 | 16.69±8.47 | 0.605 | 16.39±6.57 | 16.86±9.74 | 0.835* | 14.66±4.46 | 16.16±3.14 | 0.520 |
| LH/FSH | 2.05±1.18 | 0.94±0.71 | ＜0.001* | 2.15（0.06,5.42） | 0.82（0.26,4.37） | ＜0.001* | 1.71（0.34,5.26） | 0.68（0.28,2.11） | 0.035 |
| TT(nmol/L) | 2.27±0.76 | 1.34±0.46 | ＜0.001* | 2.25±0.76 | 1.34±0.47 | ＜0.001* | 2.32±0.76 | 1.35±0.43 | 0.001 |
| Daily steps# | 5370（570,16329） | 5467（896,13209） | 0.563 | 5105（570,16329） | 5477（896,13209） | 0.895 | 5946（1363,13676） | 2322（1311,11092） | 0.255 |
| VAI# | 1.27（0.28,6.98） | 0.82（0.33,6.15） | ＜0.001 | 1.05（0.28，3.79） | 0.75（0.33，4.04） | 0.001 | 2.03（0.55，6.98） | 2.36（0.58,6.15） | 0.734 |
| LAP# | 19.71（0.68，168.84） | 13.07（1.88,140.30） | 0.004 | 11.80（0.68，51.33） | 10.64（1.88，45.22） | 0.244 | 43.20（13.68,168.84） | 45.90（13.57,140.30） | 0.699 |
| BFP | 30.40±8.36 | 27.06±8.23 | ＜0.001 | 26.81±6.89 | 24.70±6,24 | 0.014 | 38.07±5.63 | 39.78±5.67 | 0.228 |
| VFL# | 5.00（1.00,20.00） | 4.50（1.0,20.0） | 0.775 | 3.00（1.00，12.00） | 4.00（1.00，11.00） | 0.075 | 8.00（2.50,20.00） | 12.00（4.50,20.00） | 0.045 |
| BMR(kcal) | 1258.65±136.23 | 1174.86±131.67 | ＜0.001 | 1193.36±82.70 | 1141.53±98.23 | ＜0.001 | 1399.29±122.06 | 1365.33±138.47 | 0.304 |

* Welch`s- t test; # Mann-Whitney u test. BMI, body mass index; WC, waist circumference; HC, hip circumference; WHR, waist-to-hip ratio; WHtR, waist-to-height ratio; TC, total cholesterol; TG, triglycerides; LDL, low-density lipoprotein; HDL, high-density lipoprotein; FPG, fasting plasma glucose; FINS, fasting insulin; HOMA-IR, homeostasis model assessment-estimated insulin resistance; VD, vitamin D; LH, luteinizing hormone; FSH, follicle-stimulating hormone; TT, total testosterone; VAI, visceral adiposity index; LAP, lipid accumulation product; BFP, body fat percentage; VFL, visceral fat level; BMR, basal metabolic rate.
